# Synthesis of High Molecular Weight Polyester Using in Situ Drying Method and Assessment of Water Vapor and Oxygen Barrier Properties

**DOI:** 10.3390/polym10101113

**Published:** 2018-10-09

**Authors:** Shouyun Cheng, Burhan Khan, Fahad Khan, Muhammad Rabnawaz

**Affiliations:** School of Packaging, Michigan State University, East Lansing, MI 48824-1223, USA; shouyun.cheng@sdstate.edu (S.C.); khanburh@msu.edu (B.K.); fahad438@gmail.com (F.K.)

**Keywords:** polyesters, salen chromium(III) complex, alternating ring-opening copolymerization, food packaging

## Abstract

The preparation of renewable polyesters with good barrier properties is highly desirable for the packaging industry. Herein we report the synthesis of high molecular weight polyesters via an innovative use of an in situ drying agent approach and the barrier properties of the films formed from these polyesters. High number average molecular weight (*M*_n_) semiaromatic polyesters (PEs) were synthesized via alternating ring-opening copolymerization (ROCOP) of phthalic anhydride (PA) and cyclohexene oxide (CHO) using a salen chromium(III) complex in the presence of 4-(dimethylamino)pyridine (DMAP) cocatalyst. The use of a calcium hydride (drying agent) was found to enhance the number *M*_n_ of the synthesized PEs, which reached up to 31.2 ku. To test the barrier properties, PE films were prepared by solvent casting approach and their barrier properties were tested in comparison poly(lactic acid) films. The PE films showed significantly improved water vapor and oxygen barrier properties compared to the commercial poly(lactic acid) (PLA) film that suggests the potential use of these PEs in in the food packaging industry.

## 1. Introduction

Polyesters (PEs), especially those derived from renewable resources, have attracted great interest because they are considered as an alternative to the current petrochemical-based plastics [1]. Polyesters are widely used in environmental, pharmaceutical, and commodity materials, and they are employed in packaging applications, due to their excellent barrier properties [2]. Polyesters can be produced via polycondensation or ring-opening copolymerization (ROCOP) methods [1]. The polycondensation method for polyester synthesis involves the reaction of diols with diacids (or diesters) [3]. However, this method is energy-intensive, since it requires high temperatures to induce condensation, reduced pressure to remove water or methanol and longer reaction periods to achieve high molecular weight polyesters [3].

The ring-opening copolymerization method involves a polymerization reaction between epoxides and cyclic anhydrides [4]. A benefit of ring-opening polymerizations is that they can be performed at mild temperatures without any need for the vacuum removal of the methanol/water molecules prepared during a condensation polymerization. Therefore, this reaction provides a greener synthetic approach for polyester synthesis compared to the polycondensation method. Many anhydrides and epoxides, including phthalic anhydride (PA), succinic anhydride (SA), cyclohexanedicarboxylic anhydride (CHA), maleic anhydride (MA), cyclohexene oxide (CHO), and epichlorohydrin (ECH) have been derived from renewable resources and are employed as feedstock for the ring-opening polymerization method to produce green Pes [5]. However, the ROCOP approach is not yet mature, and suffers from limitations, such as low *M*_n_ polymers and low turn-over frequency [2].

In order to increase the molecular weight of PE synthesized via the ring-opening polymerization method, many different metal-salen ligand catalysts and cocatalysts have been developed for the ROCOP of epoxides and anhydrides, including zinc [6], magnesium [7], chromium [8], cobalt [9], manganese [10], iron [11], aluminum [12], and nickel complex [13], most of which showed significantly higher activity in the presence of a nucleophilic cocatalyst, such as 4-dimethylaminopyridine (DMAP), bis(triphenylphosphine)iminium salts ([PPN]X), phosphines and ammonium salts. Duchateau and coworkers conducted a comprehensive study on the copolymerization of cyclohexene oxide (CHO) with phthalic anhydride (PA), succinic anhydride (SA), and cyclopropane-1,2-dicarboxylic acid anhydride (CAA) using salen aluminum, chromium and cobalt complexes in combination with several cocatalysts, such as bis(triphenylphosphine) iminium chloride (PPNCl) and 4-(dimethylamino)pyridine (DMAP) [14]. The highest catalytic activity was obtained by the combination of a salen chromium(III) complex catalyst with DMAP cocatalyst. The most reactive substrate was PA, which produced PEs with the highest molar mass (13 ku). Coates et al. [15] also found that salen chromium(III) complex was an effective catalyst for the ring-opening copolymerization of propylene oxide and maleic anhydride to produce high *M*_n_ poly(propylene fumarate) (17 ku). However, the *M*_n_ values of aromatic polyesters are still not sufficiently and that is the possible reasons to for not testing the barrier properties of these PE films.

A key reason for the low *M*_n_ of PE produced by ROCOP method is the presence of residual water impurity that functions as the chain transfer agent [16]. The residual water molecules originates from the reactants (i.e., epoxides and cyclic anhydrides) [14], even if they have been dried and distilled in the presence of calcium hydride. We reasoned that the incorporation of a drying agent during the polymerization could help to remove the residual water from reactants mixture, which will help to suppress undesired chain transfer reactions during the ROCOP. To the best of our knowledge, there have been no reports on the use of in situ drying agent (e.g., CaH_2_) to improve the molecular weights of aromatic/aliphatic PEs synthesized via ROCOP.

Poly(lactic acid) (PLA) is an aliphatic PE that is prepared from lactide by ring opening polymerization. PLA use in the packaging is increasing, since it is biobased, as well as compostable [17,18]. However, PLA has only moderate oxygen and water vapor permeation properties [1]. The development of bio-based PEs with improved oxygen and water vapor barrier properties for food packaging is highly desirable, since the enhanced barrier properties of packaging materials extend the shelf-lifetimes of perishable food items.

In this study, PE was synthesized via the ROCOP of CHO and PA, and then it was subsequently used to prepare films. The oxygen and water vapor permeation properties of these PE films were then compared with those of PLA films. We chose CHO and PA as precursors, because synthetic approaches exist to prepare CHO and PA from biobased triglycerides and carbohydrates, respectively [19,20]. In this research, an in situ drying agent was used for the first time during the ROCOP of CHO and PA. In the presence of calcium hydride drying agent, high *M*_n_ PEs were synthesized. These PEs were characterized via NMR, GPC, FTIR and DSC techniques. The PE with the highest *M*_n_ was employed to prepare PE films using a solvent casting method and their water vapor and oxygen barrier properties were investigated and compared to those of PLA films. To the best of our knowledge, the barrier properties of the PEs prepared by the ROCOP approach have never been reported.

## 2. Experimental Section

Materials. Cyclohexene oxide (CHO, Aldrich, St. Louis, MO, USA) was dried and distilled over calcium hydride (CaH_2,_ reagent grade > 95%, Sigma Aldrich), and then degassed via three freeze-pump-thaw cycles and stored under nitrogen in a glovebox. Phthalic anhydride (PA, Aldrich) was recrystallized from dichloromethane (DCM, Aldrich) via a literature method [16]. 4-(Dimethylamino)pyridine (DMAP, Aldrich) was used as received. Toluene (Fisher), tetrahydrofuran (THF, Aldrich) and dichloromethane were collected from the solvent purification system. The salen chromium(III) complex((R,R)-*N*,*N*′-bis(3,5-di-*tert*-butylsalicylidene)-1,2-cyclohexanediaminochromium(III) chloride, C_36_H_52_ClCrN_2_O_2_, Aldrich) was used as received. Poly (96% L-lactic acid) (PLA) with a *M*_n_ of 112.54 ku was purchased from NatureWorks LLC (Minnetonka, MN, USA) and used for film formation.

ROCOP reaction of CHO and PA. An 8 mL vial equipped with a magnetic stirring bar was charged with CHO (5 or 9 mmol), PA (5 mmol), salen Cr catalyst (10 μmol), DMAP cocatalyst (10 μmol), toluene (0.9 mL) and CaH_2_ (0 mmol or 2 mmol). The vial was then placed into a preheated aluminum heating block in the glove box. The copolymerization reaction was performed at 110 °C. After the desired polymerization time of 22 h, an aliquot of the crude reaction mixture was collected for ^1^H NMR analysis to determine the conversion degree of CHO. The rest of the reaction mixture was diluted with dry THF (2 mL), filtered through a 0.45 μm PTFE syringe filter (Acrodisc) to remove CaH_2_, and precipitated from hexane (20 mL). The obtained PEs were centrifuged and dried in a rotary evaporator at room temperature (20 °C) for 2 h until a constant weight reached.

PE and PLA film preparation. PE and PLA were used to prepare films (Table 1 and Table S1 (Supplementary Materials)) for the barrier properties assessment using the following procedure. The 200 mg of the selected polymer (PE and PLA) was dissolved in THF (0.4 mL). This solution was then cast onto an aluminum pan and covered by a glass jar to avoid the fast evaporation of THF and get films without defects and any perforations. Once the films were visibly dry (after 12h at room temperature), the pans containing the PE or PLA films were shifted to an oven at 60 °C for 1 h to remove the residual THF. The pans were subsequently cooled to room temperature and the films were then detached from the pan, which were proceeded for evaluation of their water vapor and oxygen barrier properties.

Molecular weights of the prepared PEs were determined using a Waters Breeze SEC (Size exclusion chromatography) RI (Refractive Index) chromatographic system (Waters 1515 pump, Waters 2410 refractive index detector and Waters 717plus Autosampler). GPC (Gel Permeation Chromatography) analysis were performed using four Styragel columns (HR1, HR2, HR3, and HR4) at 35 °C in THF at an elution rate of 1 mL·min^−1^. Calibrations were performed using monodisperse polystyrene standards. As the GPC is a relative analytical tool to get the molecular weights, therefore all the polyesters were characterized under the same GPC conditions, using the same GPC method and GPC instrument. This enabled us to confidently compare the *M*_n_ and Ð values for different polyesters.

Differential scanning calorimetry (DSC) analysis of the PEs was conducted in a Q100 differential scanning calorimeter (TA Instruments, New Castle, DE, USA) equipped with a mechanical cooling system.

The absorbance of the PEs was recorded in an Iraffinity-1S Fourier-transform infrared spectroscopy (FTIR, Shimadzu, Japan) instrument. The spectra were recorded over a range from 500 to 4000 cm^−1^. FTIR method mode is ATR. The polyester powder sample was put in the diamond tip and the pressed sample spectra was taken in ATR mode. The FTIR background signal was removed before sample FTIR analysis.

Water vapor and oxygen barrier properties. Water vapor and oxygen barrier performance of the film samples were measured using Permatran^®^ model 3/33 and Ox-tran^®^ model 2/21 permeation testing instruments (Mocon, Minneapolis, MN, USA), respectively. The film samples were mounted on aluminum foil masks with an exposed area of 0.18 cm^2^. Samples were tested at 50% relative humidity (RH) and 20 °C.

## 3. Results and Discussion

**Scheme 1:**
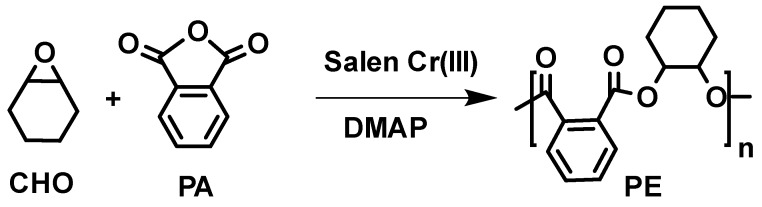
Ring-opening copolymerization (ROCOP) reaction between cyclohexene oxide (CHO) and PA in the presence of salen Cr(III) and -(dimethylamino)pyridine (DMAP).

**Figure 1:**
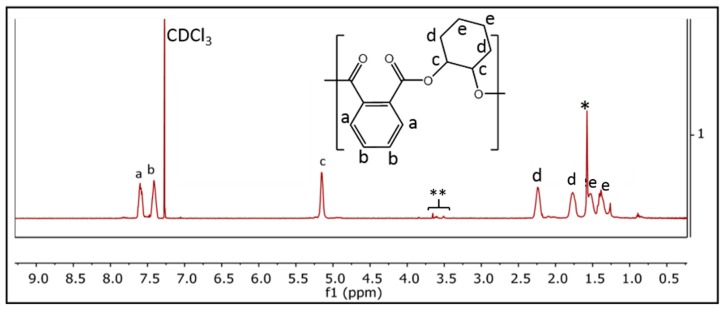
Representative ^1^H NMR spectrum of the PE (entry#3 in Table 2). * represents water impurity in the CDCl_3_ solvent, ** represents ether linkage in the PE.

Scheme 1 Illustrates the PE synthesis via the ROCOP of CHO and PA in the presence of salen Cr(III) catalyst and DMAP cocatalyst. A detailed proposed mechanism is provided in the supporting information. In certain cases, CaH_2_ was added during polymerization as a drying agent. The synthesized PE was characterized by ^1^H NMR (Figure 1), which showed four broad singlet peaks in the region of 1.13–2.16 ppm corresponding to four chemically different protons (axial and equatorial) of the cyclohexane ring. Similarly, the characteristics peak at 5.2 ppm was assigned to C(O)OCH- protons, while the aromatic protons of the PA appeared between 7.4–7.6 ppm. In addition, the signal integrations of the two protons of C(O)OCH- and the four aromatic protons of the PA is essentially a 1:2 ratio, suggesting that ~1:1 ratio of CHO and PA in the polyester. Similar assigning signals to specific protons of NMR spectra for polyester was reported in the study of Mundil et al. [11]. There are very weak signals in the ether linkage region (3.6 ppm) in the ^1^H NMR spectrum (marked as “**”) of PE, which corresponds to the ether linkage formed by the two consecutive insertions of cyclohexene oxide during the polymerization. The very weak signals of the ether linkage in the ^1^H NMR spectrum reveals that there is very little insertion of the consecutive cyclohexanone rings in the main alternating PE backbone. The catalytic mechanism of cyclohexene oxide (CHO) and phthalic anhydride (PA) copolymerization reaction using Salen Cr and -(dimethylamino)pyridine (DMAP) catalysts is shown in Scheme S1 (Supplementary Materials).

The preliminary tests of drying all the reagents were conducted to synthesize the polyester. However, the *M*_n_ of the polyester (5–6 ku) is much lower than the polyester synthesized in the presence of calcium hydride. Therefore, we conducted the test of synthesizing the polyester in the presence of both drying and calcium hydride. The ROCOP reaction between CHO and PA was investigated at different CHO/PA ratios (1.0:1.0 and 1.8:1.0). The properties of the resultant PEs are summarized in Table 2. The CHO/PA ratio showed a significant influence on the CHO conversion ratio and the *M*_n_ of the prepared PEs. A high CHO conversion ratio of 96.2% was obtained at the CHO/PA ratio of 1.0:1.0 in the absence of CaH_2_. This CHO conversion ratio decreased to 67.9% with an increased CHO/PA ratio (1.8:1.0) in the absence of CaH_2_. However, the *M*_n_ of the PE increased significantly to 13.2 ku at a CHO/PA ratio of 1.8:1.0 compared to the value of 5.8 ku that was obtained at a CHO/PA ratio of 1.0:1.0. A similar trend has also been observed by Saini et al. [21].

The *M*_n_ of the PE increased significantly in the presence of CaH_2_. For instance, in presence of CaH_2_, the *M*_n_ of PE increased to 31.2 and 20.8 ku at the CHO/PA ratios of 1.0:1.0 and 1.8:1.0, respectively. This increase in the M_n_ possibly corresponds to the removal of water impurity by CaH_2_ and the suppression of subsequent chain transfer inhibitions. As shown in Table 2, all four of the synthesized PEs obtained exhibited a low polydispersity (1.06–1.10).

The thermal properties of these PEs were also investigated. It was found that the PEs exhibited enhanced thermal properties when their M_n_ values increased. For example, the *T*_g_ of the PEs was elevated from 75 (entry 1) to 121 °C (entry 2) as PE proceeded from the lowest to the highest *M*_n_ of 5.8 and 31.2 ku, respectively. The *T*_g_ values of the PE reported in entry 3 and 4 were also very high (~110 °C). These high *T*_g_ values for the Pes, shown in entries 2–4 of Table 2, suggest their potential for packaging use. The *T*_g_ of the PE in entry 3 was slightly increased relative to entry 4 PE despite entry 4 PE has high *M*_n_. This anomaly is probably attributed to the high conversion of CHO (77.5%) in the PE of entry 4 compared to 67.92% in entry 3 PE. As CHO is flexible relative to PA, that’s explain why the *T*_g_ was a bit lower for the entry 4 PE despite high *M*_n_.

The representative FTIR spectra of the PEs prepared in the presence and absence of CaH_2_ are shown in Figure S1 (Supplementary Materials). The FTIR spectrum of the PE confirms the ester linkages. For example, the band corresponding to the symmetric stretching vibration of the ester carbonyl (C=O) group appeared at 1720 cm^−1^ [22]. Meanwhile, the bands at 1250 and 1066 cm^−1^ corresponds to the asymmetric and symmetric stretching vibrations of the C–O–C group, respectively [11].

**Figure 2:**
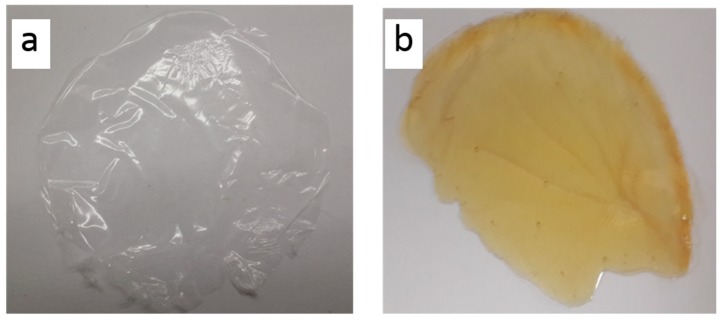
Images of PE and PLA films: (**a**) PLA; (**b**) PE.

Barrier properties of the PE and Poly(lactic acid) (PLA) films. A key goal of this study is to demonstrate the barrier properties of the PEs prepared by ROCOP approach. The PE sample with a *M*_n_ of 31.2 ku was used to prepare films for barrier performance tests. We chose this PE because of its high *M*_n_. Representative images of the PE and PLA films used in this study are shown in Figure 2 and Figure S2 (Supplementary Materials). PLA film (Figure 2a) was used prepared as reference in this study.

The above films were subjected to water vapor and oxygen permeability tests. These tests were conducted because water vapor and oxygen barrier properties are the most significant factors that affect quality and shelf-life of a product [23]. The results obtain from the oxygen and water vapor permeabilities of the PE and PLA films are shown in Table 3 and Figures S3 and S4 (Supplementary Materials). The oxygen permeability of the PE film was 544 cc-mil/(m²-day-atm), which was considerably lower than that of the PLA film 1528 cc-mil/(m²-day-atm). This 62% reduction in oxygen permeability observed for the PE film compared to PLA could be attributed to relatively strong secondary interaction strengthened by the presence of aromatic benzene rings on PE chains as compared to PLA, which should theoretically have weak secondary interactions. The water vapor permeation of the PE film was 62 g-mil/(m²-day-atm), which was significantly lower compared to that of the PLA film (168 g-mil/(m²-day-atm)). This 63% reduction in the water permeability of the PE films relative to PLA films is, again, probably due to the strong secondary interactions and closest chain packing of PE. Thus, the obtained PE films have shown a significant improvement in the water and oxygen barrier properties, which will provide these materials applications in the food packaging industry to protect perishable goods against oxygen and moisture degradation [24]. The water vapor and oxygen permeability of the PE (0.18 mm) was compared to another PLA film with similar thickness (0.19 mm). The water vapor and oxygen permeability the PE were still much lower than that of PLA film with similar thickness.

The lower content of residual CaH_2_ in the obtained polymers is important. Therefore, after the polyester synthesis, the reaction mixture was diluted with dry THF (2 mL), filtered through a 0.45 μm PTFE syringe filter to remove CaH_2_ and residual catalysts, and then precipitated from hexane (20 mL). The filtration and precipitation removed most of the CaH_2_ and residual catalysts. Besides, the residual CaH_2_ and catalysts could be further removed through filtration by silica or celite in the future studies. The obtained polyester was then cleaned through centrifuge and drying in a rotary evaporator at room temperature (20 °C) for 2 h to remove the residual solvents, such as THF and hexane. However, more repetitions of dissolving the polyester to THF, filtration through PTFE syringe filter and celite/silica, centrifuge and drying will be conducted to further clean polyester and remove the CaH_2_ or residual catalyst left in the final polyester.

The exposure to chromium compounds, such as ions Cr^3+^ and its organic ligands could result in the formation of ulcers, which will persist for months and heal very slowly [25]. The possible leaking chromium compounds in the food/cosmetic packaging might have this negative effect. However, the effect of chromium compound is limited, since only small ratio of chromium (salen Cr:DMAP:PA = 1:1:500) was used in polyester synthesis in this study. Most of the chromium catalyst was also removed during precipitation, washing, and filtration.

The thickness of the films used in a real application is around 2–3.2 mm, which is similar to PLA film [26]. The 2–3.2 mm thickness of synthesized PEs could be made using casting method. Therefore, no obvious thresholds for the film thickness in case of the synthesized PEs was found yet. The PE film is stable in the drying temperature of 60 °C for 1 h, and it should be relatively stable, due to its relatively high *T*_g_ of 121 °C.

## 4. Conclusions

We demonstrated for the first time the in situ use of a drying agent to improve the *M*_n_ of a PE. Aromatic polyesters (PEs) were synthesized via an in situ drying ROCOP reaction between CHO and PA using salen chromium(III) as a catalyst and 4-(dimethylamino)pyridine (DMAP) as a cocatalyst in the presence of CaH_2_. The use of a drying agent significantly increased the *M*_n_ of the PEs. The NMR and FTIR spectra confirmed the chemical structure of PE. The highest *M*_n_ of 31.2 ku was synthesized at CHO/PA/CaH_2_ of 1:1:0.4. The *T*_g_ of this high *M*_n_ PE was as high as 121 °C. We tested the barrier properties of a polymer prepared via the ROCOP approach for the first time. The barrier properties of the highest *M*_n_ PE were evaluated against those of PLA. The PE films showed significant improvement in the barrier performance against oxygen and water vapor than was provided by PLA, which will encourage the possible use of these PE films for food packaging. The PE films also showed better oxygen and water vapor barrier performance than the PE and PLA mixture films. The negative effect of chromium compound is limited, due to the small ratio of chromium (salen Cr:DMAP:PA = 1:1:500) used in polyester synthesis.

## Figures and Tables

**Table 1:** Films fabricated from polyester (PE), poly(lactic acid (PLA) or PLA-PE mixtures.

Samples	PLA (mg)	PE (mg)	THF
1	200	0	0.4 mL
2	0	200	0.4 mL

PE characterization. ^1^H NMR spectra of the PE samples were recorded with a 500 MHz NMR spectrometer (Agilent, Santa Clara, CA, USA) using chloroform-*d* (CDCl_3_) as the solvent.

**Table 2:** Properties of PEs synthesized from CHO and PA in the presence of salen Cr and DMAP ^a^.

Entry	CHO/PA/CaH_2_	CHO Conversion ^b^ (%)	PE		
			*M*_n_^c^ (ku)	Ð ^c^	*T*_g_^d^ (°C)
1	1:1:0	96.2	5.8	1.07	75
2	1:1:0.4	99.0	31.2	1.06	121
3	1.8:1:0	67.9	13.2	1.08	113
4	1.8:1:0.4	77.5	20.8	1.10	110

^a^ T = 110 °C; V_toluene_ = 0.9 mL; salen Cr:DMAP:PA = 1:1:500; ^b^ Determined by ^1^H NMR spectroscopy; ^c^ *M*_n_ (number average molecular weight) and Ð (polydispersity) determined by SEC; ^d^ Determined by differential scanning calorimetry (DSC).

**Table 3:** Permeability values for PLA and PE films.

Sample	Thickness (mm)	Oxygen Permeability (cc. mil/m^2^-day-atm)	Water Vapor Permeability (g. mil/m^2^-day-atm)
PLA	0.04	1528 ± 593.88	168 ± 62.03
PLA	0.19	1508 ± 565.42	148 ± 12.10
PE	0.18	544 ± 145.00	62 ± 5.01

## Supplementary Materials

The following are available online at http://www.mdpi.com/2073-4360/10/10/1113/s1, Scheme S1: Catalytic mechanism cyclohexene oxide (CHO) and phthalic anhydride (PA) copolymerization reaction using Salen Cr and -(dimethylamino)pyridine (DMAP) catalysts, Figure S1: Representative 2. Fourier-transform infrared spectroscopy (FTIR) spectra of the polyester (PE) (entry 1 and 2 in Table 2), Table S1: Two films fabricated from poly(lactic acid) (PLA)-PE mixtures, Figure S2: Images of all the PE and PLA films (a- PLA; b- PLA:PE (3:1); c- PLA:PE (1:1); d- PE), Figure S3: Oxygen barrier properties of all the PE and PLA films, Figure S4: Water vapor barrier properties of all the PE and PLA films.
