# Supplementary material for: Synthesis of High Molecular Weight Polyester Using in Situ Drying Method and Assessment of Water Vapor and Oxygen Barrier Properties
Source: Polymers (Basel). 2018 Oct 9;10(10):1113. doi: 10.3390/polym10101113 (PMC6403584; doi:10.3390/polym10101113)
Supplement: Supplementary file 1 [file polymers-10-01113-s001.pdf]

Supporting information:

Synthesis of High Molecular Weight Aromatic Polyester Using in Situ Drying Agent and the Assessment of Their Water Vapor and Oxygen Barrier Properties

Shouyun Cheng, Burhan Khan, Fahad Khan and Muhammad Rabnawaz *

School of Packaging, Michigan State University, 448 Wilson Road East Lansing, MI 48824-1223, USA; shouyun.cheng@sdstate.edu (S.C.); khanburh@msu.edu (B.K.); fahad438@gmail.com (F.K.)

* Correspondence: rabnawaz@anr.msu.edu; Tel.: +1-517-432-4870

1. Mechanism of ROAC using salen Cr and DMAP catalyst

The catalytic mechanism of cyclohexene oxide (CHO) and phthalic anhydride (PA) copolymerization reaction, using Salen Cr and -(dimethylamino)pyridine (DMAP) catalysts is shown in Scheme S1. Salen Cr complex serves as a Lewis acid catalyst for epoxide binding/activation, and opens the epoxide ring in the presence of DMAP [1]. In chain initiation process, CHO was activated by Salen Cr and the ring was opened by the Cl^{-1} of Salen Cr/DMAP pairs or by the Cl^{-1} Salen Cr/CHO. Then an alkoxide intermediate was generated with an amine initiator in one end and Cl^{-} in the other end. Subsequently, the active alkoxide selectively reacted with an anhydride to form an ester unit. In the chain propagation process, Salen Cr continually worked to activate and open the ring of CHO [1].

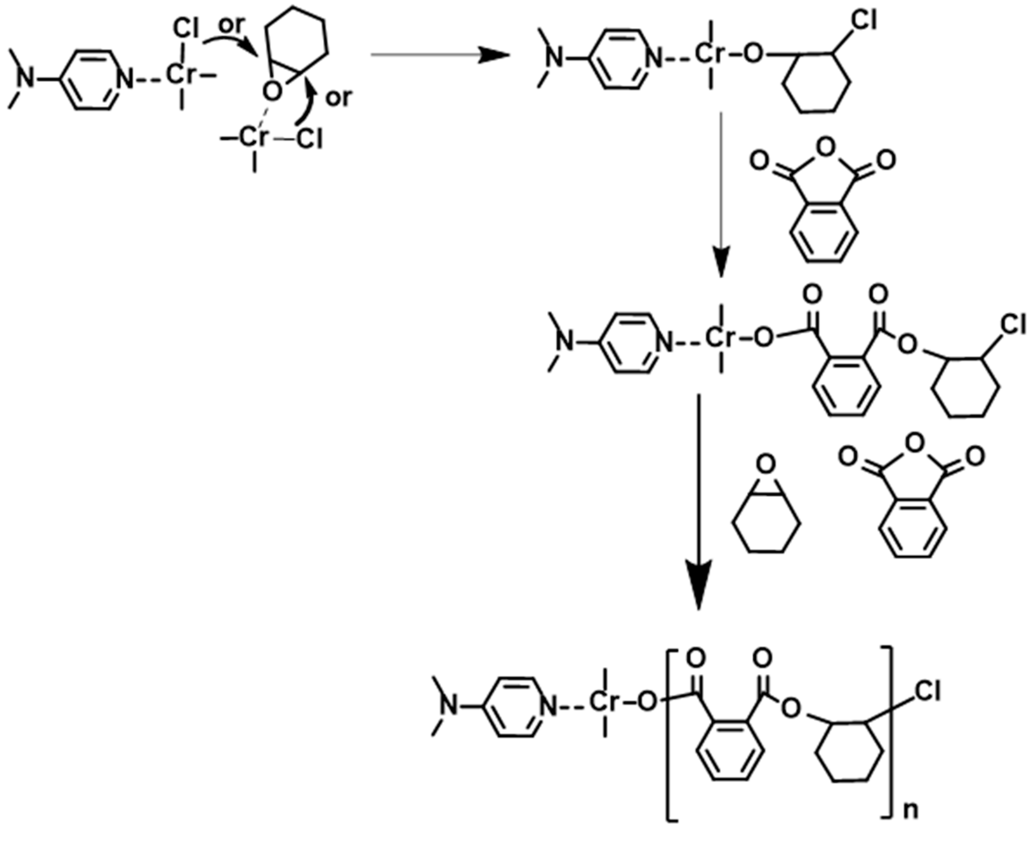

Scheme S1. Catalytic mechanism of cyclohexene oxide (CHO) and phthalic anhydride (PA) copolymerization reaction using Salen Cr and -(dimethylamino)pyridine (DMAP) catalysts.

2. Fourier-transform infrared spectroscopy (FTIR) spectra of the polyester (PE)

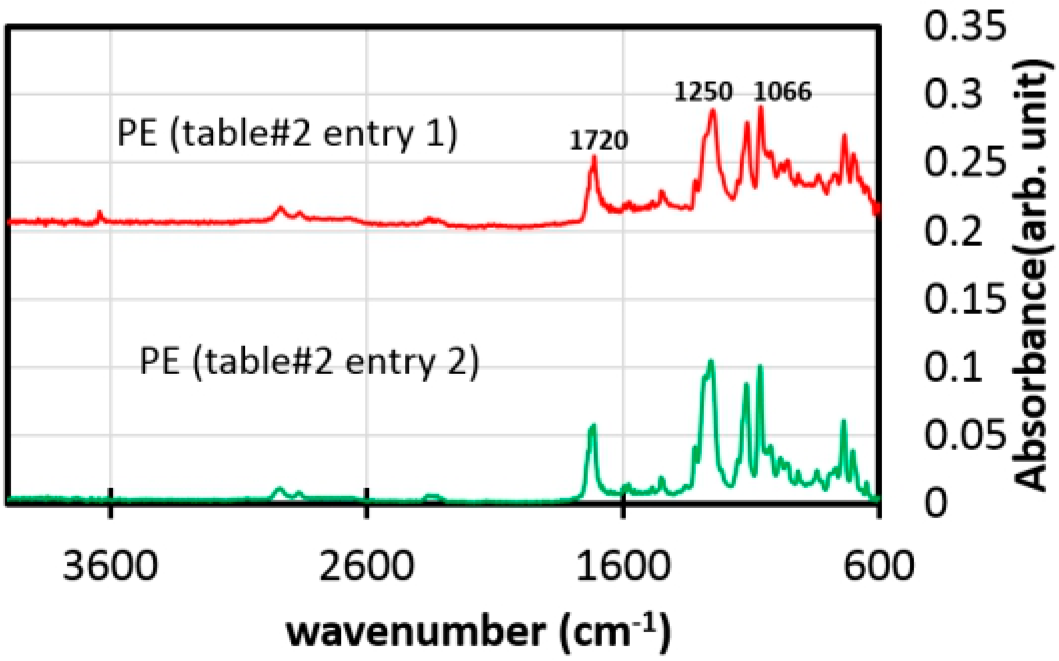

Figure S1. Representative 2. Fourier-transform infrared spectroscopy (FTIR) spectra of the polyester (PE) (entry 1 and 2 in Table 2).

3. Films prepared from poly(lactic acid) (PLA)-PE mixtures

Table S1. Two films fabricated from poly(lactic acid) (PLA)-PE mixtures.

Samples	PLA (mg)	PE (mg)
1	150	50
2	100	100

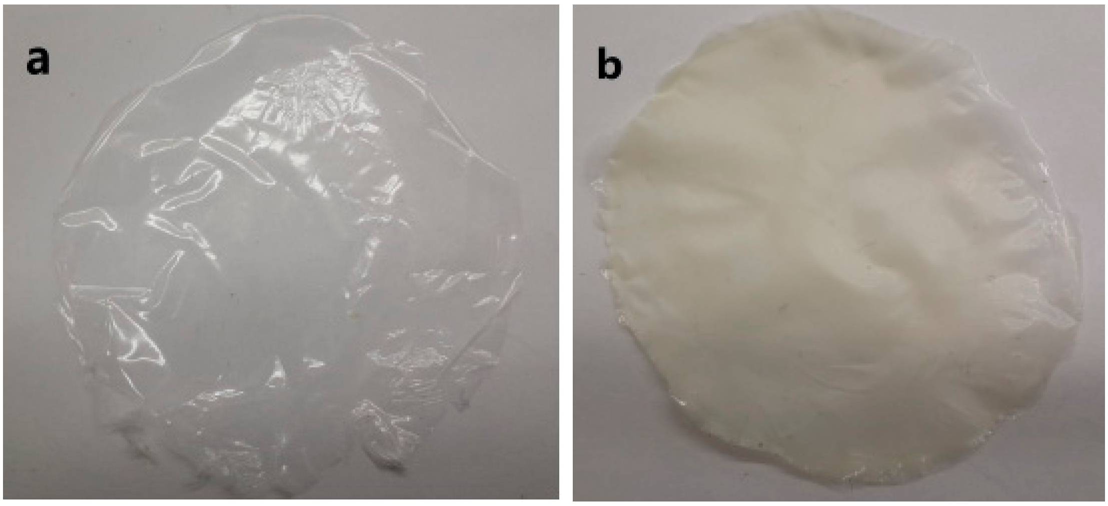

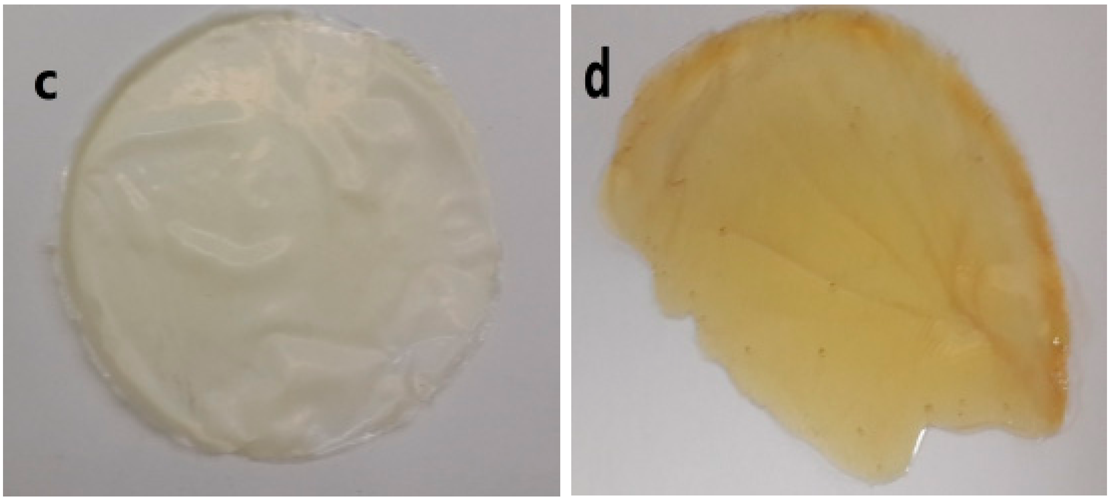

Figure S2. Images of all the PE and PLA films (a- PLA; b- PLA:PE (3:1); c- PLA:PE (1:1); d- PE).

4. Oxygen and water vapor permeabilities for films of PLA-PE mixtures

The comparison of oxygen and water vapor permeabilities for all the PE and PLA films are shown in Figure S3 and Figure S4, respectively. The oxygen permeability of the PE film was 544 cc - mil / (m^2 - day - atm), which was considerably lower than that of the PLA film 1528 cc - mil / (m^2 - day - atm). The water vapor permeation of the PE film was 62 gm - mil / (m^2 - day - atm), which was significantly lower compared to that of the PLA film (168 gm - mil / (m^2 - day - atm)). However, the films obtained from the PE and PLA mixture showed poor oxygen and water vapor permeation compared to PLA and PE films. This result may have been due to the phase separation between the PLA and PE components in the matrix [2].

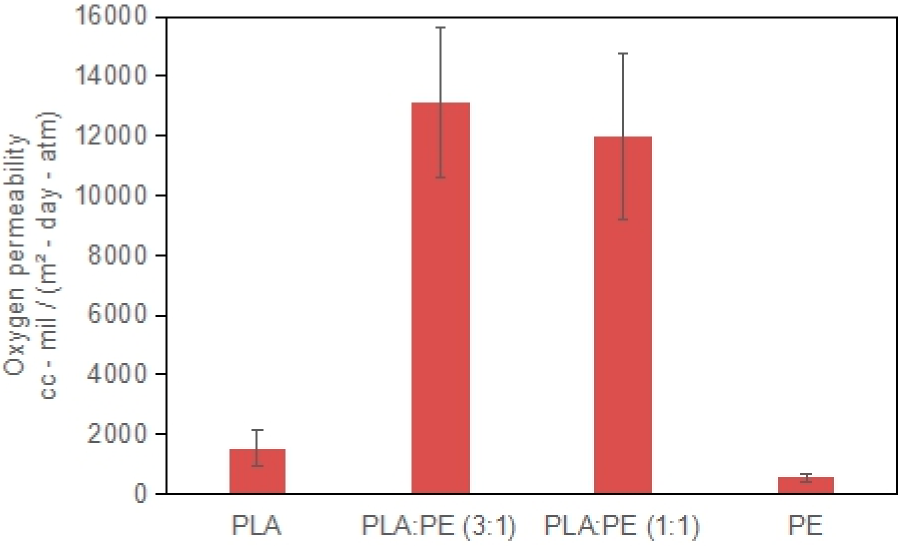

Figure S3. Oxygen barrier properties of all the PE and PLA films.

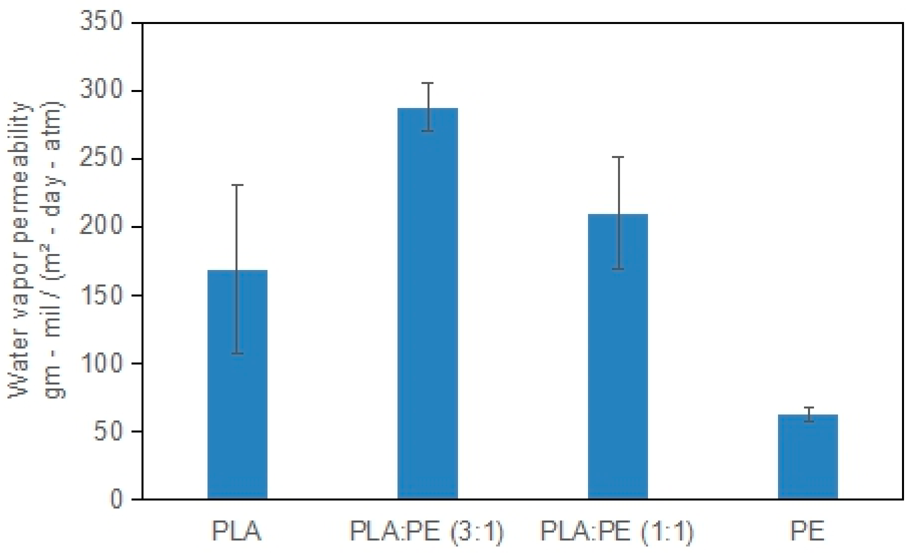

Figure S4. Water vapor barrier properties of all the PE and PLA films.

References

1. D. J. Darensbourg, R. R. Poland and C. Escobedo, *Macromolecules*, **2012**, *45*, 2242-2248.
2. K. Majdzadeh-Ardakani, S. Zekriardehani, M. R. Coleman and S. A. Jabarin, *International Journal of Polymer Science*, 2017, **2017**.
